# Systematic Analysis on the Effect of Sintering Temperature for Optimized Performance of Li_0.15_Ni_0.45_Zn_0.4_O_2_-Gd_0.2_Ce_0.8_O_2_-Li_2_CO_3_-Na_2_CO_3_-K_2_CO_3_ Based 3D Printed Single-Layer Ceramic Fuel Cell

**DOI:** 10.3390/nano11092180

**Published:** 2021-08-25

**Authors:** Muhammad Imran Asghar, Pyry Mäkinen, Sini Virtanen, Anna Maitre, Maryam Borghei, Peter D. Lund

**Affiliations:** 1 New Energy Technologies Group, Department of Applied Physics, School of Science, Aalto University, P.O. Box 15100, FI-00076 Espoo, Finland; pyry.makinen@aalto.fi (P.M.); sini.n.virtanen@aalto.fi (S.V.); anna.maitre@aalto.fi (A.M.); peter.lund@aalto.fi (P.D.L.); 2 Faculty of Physics and Electronic Science, Hubei University, Wuhan 430062, China; 3 Department of Bioproducts and Biosystems, School of Chemical Engineering, Aalto University, FI-00076 Espoo, Finland; maryam.borghei@aalto.fi

**Keywords:** 3D printing, ceramic, fuel cell, mixed ionic and electronic conductivity, nanocomposite, single-layer, sintering, reaction kinetics

## Abstract

Single-layer ceramic fuel cells consisting of Li_0.15_Ni_0.45_Zn_0.4_O_2_, Gd_0.2_Ce_0.8_O_2_ and a eutectic mixture of Li_2_CO_3_, Na_2_CO_3_ and K_2_CO_3_, were fabricated through extrusion-based 3D printing. The sintering temperature of the printed cells was varied from 700 °C to 1000 °C to identify the optimal thermal treatment to maximize the cell performance. It was found that the 3D printed single-layer cell sintered at 900 °C produced the highest power density (230 mW/cm^2^) at 550 °C, which is quite close to the performance (240 mW/cm^2^) of the single-layer cell fabricated through a conventional pressing method. The best printed cell still had high ohmic (0.46 Ω·cm^2^) and polarization losses (0.32 Ω·cm^2^) based on EIS measurements conducted in an open-circuit condition. The XRD spectra showed the characteristic peaks of the crystalline structures in the composite material. HR-TEM, SEM and EDS measurements revealed the morphological information of the composite materials and the distribution of the elements, respectively. The BET surface area of the single-layer cells was found to decrease from 2.93 m^2^/g to 0.18 m^2^/g as the sintering temperature increased from 700 °C to 1000 °C. The printed cell sintered at 900 °C had a BET surface area of 0.34 m^2^/g. The fabrication of single-layer ceramic cells through up-scalable 3D technology could facilitate the scaling up and commercialization of this promising fuel cell technology.

## 1. Introduction

**Figure 1:**
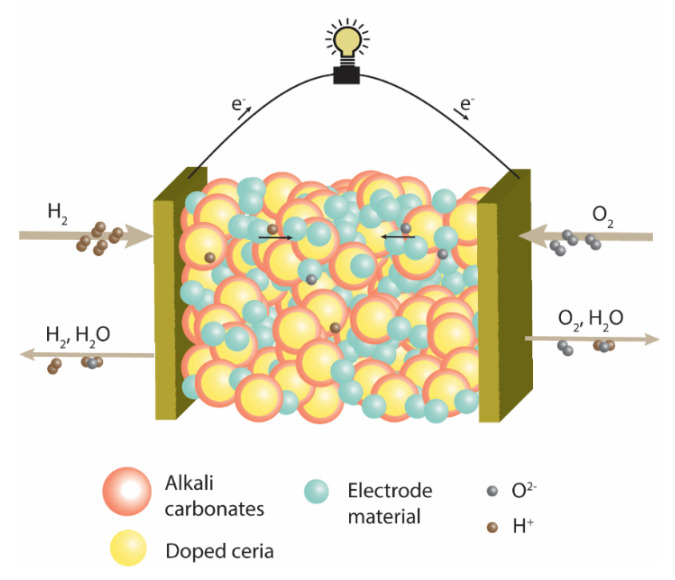
Schematic of working principle of a single-layer ceramic fuel cell.

Single-layer ceramic fuel cells are emerging as a potential fuel cell technology. In a short span of time since their invention in 2011 [1,2], these fuel cells have been reported to have remarkably high performance (up to 1 W/cm^2^) at a low temperature (550 °C) [3,4,5]. Unlike a tradition three-layer ceramic fuel cell, in which distinct anode, electrolyte and cathode layers function either as an electrode or electrolyte, a single-layer ceramic fuel cell consists of only a single layer that supports the required oxidation–reduction reactions and the ionic transport in the cell. Basically, a single-layer cell consists of a homogenous mixture of an ionic conductor and an electrode material [6,7,8]. During the operation of a single-layer cell, a fuel and an oxidant are supplied on the opposite sides of the cell. As a result of redox reactions, O^2−^ and H^+^ are generated, which travel through the layer. Interestingly, the electrons generated at one side of the cell do not travel through the cell and the layer operates as a fuel cell. To explain the avoidance of short-circuiting in novel fuel cell technology, various hypotheses have been provided in the literature [3,7,8,9], such as the heterojunction, Schottky or PN-junction principle. However, more investigation is needed to better understand the cause of electronic blockage in the cell. A schematic structure of a single-layer ceramic fuel cell is shown in Figure 1, illustrating the operation of the cell.

Various materials have been reported in single-layer ceramic fuel cells. Ionic conductors used in single-layer cells are mostly based on pure doped ceria, and composites of doped ceria and alkali carbonates. The electrode materials used in single-layer cells include LiCoO_2_-LiFeO_2_ [10], Ni_0.8_Co_0.15_Al_0.05_LiO_2_ [11], La_0.6_Sr_0.4_Co_0.2_Fe_0.8_O_3-δ_ [12], CuFe_2_O_4_ [13,14], Li_0.15_Ni_0.25_Cu_0.1_Zn_0.2_Fe_0.3_O_x_ [15], Li_0.3_Ni_0.6_Cu_0.07_Sr_0.03_O_2-δ_ [16], Sr_2_Fe_1.5_Mo_0.5_O_6-δ_ [17], Li_0.15_Ni_0.45_Zn_0.4_O_2_ [18], etc. In addition, proton conductors (BaCe_0.7_Zr_0.1_Y_0.2_O_3-δ_) have been used in single-layer cells [19]. Although single-layer cells have been reported as having high performance, we observed that the studies mostly utilized Ni_0.8_Co_0.15_Al_0.05_LiO_2_ (NCAL)-coated Ni foams as the “current collector”. In our previous study [9], we showed that NCAL-coated Ni foams acted as electrodes when applied on the sides of the cells, making them similar to a conventional three-layer cell structure. Removing NCAL-coated Ni foam reduced the performance of single-layer cells by more than half [9].

The key challenge to optimize the performance of a true single-layer fuel cell is to find the appropriate density of the single layer. If the single layer is too dense, it would have higher polarization losses because of inefficient electrode processes, mainly due to a low concentration of triple-phase boundaries (TPBs). On the other hand, if the single layer is too porous, there is a danger of gas crossover and poor ionic conduction in the cell. In our earlier study [14], we systematically studied the effect of sintering temperature on the porosity of the single layer and the cell performance. We found that pressed single-layer cells produced the best result when sintered at 700 °C [14]. In most of the single-layer ceramic fuel cell studies reported in the literature, the cells were also sintered at 700 °C [2,9,20,21,22].

Single-layer ceramic fuel cells have been mainly fabricated with the pressing method, which is commonly used for lab-scale ceramic fuel cell research. In this fabrication method, the fuel cell material is pressed with a high pressure (e.g., 250 MPa) for a certain time (e.g., 2 min). For instance, pressing a 13 mm diameter cell with a pressure of 250 MPa requires a load of 3.4 tons. However, to produce a larger cell (>100 mm diameter), the load needs to be greater than 200 tons, which is not practical for fuel cell production. Furthermore, with the pressing method, it is hard to precisely control the film thickness of large cells. Therefore, the pressing method is not generally considered suitable for large-scale production of this technology. To bring this potential fuel cell technology closer to commercialization, there is a need to fabricate these devices with suitable up-scalable technologies such as printing methods. Digital manufacturing has been recently introduced to fabricate ceramic fuel cells [23,24,25,26,27,28,29,30,31,32,33,34,35,36,37]. Digital manufacturing offers various advantages over conventional printing techniques such as screen printing [38,39] and tape casting [40,41]. For instance, the production cost of fuel cells is expected to be decreased by preventing material loss and by reducing the number of fabrication steps involved in device fabrication. Furthermore, with digital printing, it is possible to design new shapes that may help to improve the electrochemical performance of the cell and reduce the thermal stresses in the cell, improving its long-term stability. On the contrary, conventional printing techniques restrict the shape of the cell architecture to planner and tubular designs. Several digital printing technologies, including direct inkjet printing [23,24], selective laser sintering [25,26], stereolithography [27,28], digital light processing [29,30], robocasting or extrusion (also known as direct writing) [31,32,33,34], a hybrid of different technologies [35,36,37] etc., have been used to fabricate the anode, electrolyte or cathode of a three-layer ceramic fuel cells. Among these technologies, extrusion-based 3D printing is one of the most promising technologies to fabricate single-layer ceramic fuel cells with the least material waste, and it is employed in this work.

For 3D printing of a single-layer ceramic fuel cell, Li_0.15_Ni_0.45_Zn_0.4_O_2_-Gd_0.2_Ce_0.8_O_2_-Li_2_CO_3_-Na_2_CO_3_-K_2_CO_3_ was chosen as the composite material [9,21,22]. Since porosity is one of the most important factors to improve a single-layer cell’s performance and long-term stability, we focused on finding an appropriate thermal treatment for the printed single-layer ceramic cell that produces the optimal porosity in the cell. This is the first study to the best of our knowledge where a complete single-layer ceramic fuel cell is fabricated through an extrusion-based 3D printing technology.

## 2. Materials and Methods

**Figure 2:**
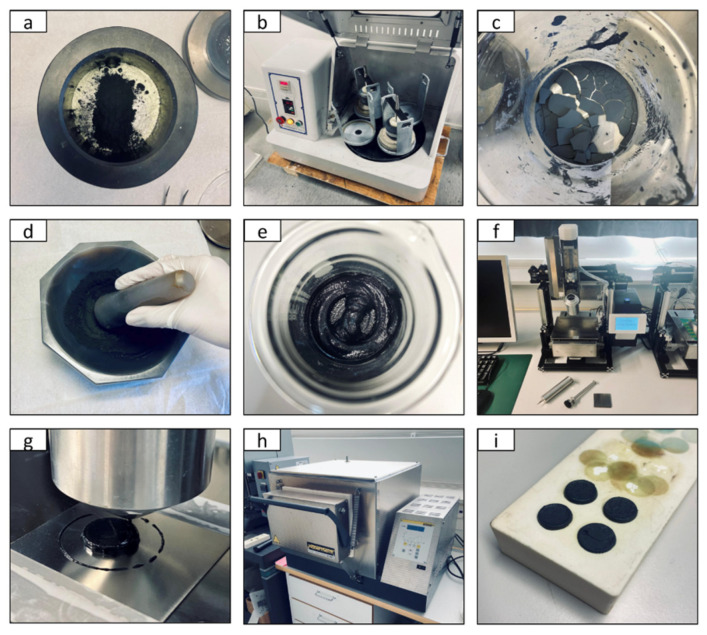
The steps involved in the fabrication of a single-layer ceramic fuel cell through extrusion-based 3D printer. (**a**) Ionic conductor and electrode powders in a milling jar, (**b**) mixing of powders in a ball milling machine using acetone solvent, (**c**) drying of the powders after ball milling step, (**d**) grinding of powders to fine particles, (**e**) preparation of paste of the composite material, (**f**) extrusion-based 3D printer used for fabrication of cells, (**g**) 3D printing of single-layer ceramic nanocomposite fuel cell, (**h**) oven used for sintering printed cells, (**i**) cells with appropriate density after the sintering process.

The chemicals and their precursors were purchased from Sigma-Aldrich. To prepare the ionic conductor for single-layer cells, gadolinium-doped ceria that contains 20 mol% gadolinium as the dopant was mixed with a eutectic mixture of lithium carbonate, sodium carbonate and potassium carbonate (Gd_0.2_Ce_0.8_O_2_ 70wt%, eutectic mixture of Li_2_CO_3_, Na_2_CO_3_ and K_2_CO_3_ 30wt%) in a planetary ball milling machine at 200 rpm for 1 h using acetone as a solvent. The mixture was first dried at room temperature for almost 24 h and later calcined at 700 °C for 2 h. Li_0.15_Ni_0.45_Zn_0.4_O_2_ was chosen as an electrode material for single-layer cells, which was synthesized by first mixing lithium carbonate, nickel carbonate basic hydrate and zinc nitrate hexahydrate in a molar ratio of 3:9:8 (Li:Ni:Zn). Later, the powders were mixed in a planetary ball milling machine at 200 rpm for 1 h using acetone as a solvent. The mixture was first dried at room temperature for almost 24 h and later calcined at 800 °C for 3 h. After the successful synthesis of the ionic conductor and electrode material, the materials (Gd_0.2_Ce_0.8_O_2_-Li_2_CO_3_-Na_2_CO_3_-K_2_CO_3_ 60wt%, Li_0.15_Ni_0.45_Zn_0.4_O_2_ 40wt%) were placed in a jar as shown in Figure 2a. The materials were again mixed in a planetary ball milling machine at 200 rpm for 1 h using acetone as a solvent, as shown in Figure 2b. The mixture was dried at room temperature for 24 h and calcined for 2 h at 700 °C, as shown in Figure 2c. Then, the mixture was ground to a fine powder by using a mortar and pestle, as shown in Figure 2d. After this step, the composite powder was ready for single-layer cell fabrication.

In order to fabricate fuel cells through a printing method, first, a terpinol-based paste of the composite material (Li_0.15_Ni_0.45_Zn_0.4_O_2_-Gd_0.2_Ce_0.8_O_2_-Li_2_CO_3_-Na_2_CO_3_-K_2_CO_3_) with appropriate rheological properties was developed, as shown in Figure 2e. The paste consisted of terpinol (60 wt%) and the composite material (40 wt%). No additional binders or plasticizers were added to the paste. The viscosity of the paste was measured using a sine-wave vibro viscometer (SV-10) and was found to be 2.4 Pa.s at 23.1 °C. The single-layer ceramic fuel cells were fabricated using the extrusion-based 3D printer shown in Figure 2f. To print the composite material, first, the printable paste (10 g) was loaded in a steel syringe of 22 mm in diameter and inserted inside the printhead. The precise x–y–z axis motion of the printhead was controlled through stepper motors. A stepper motor-controlled piston was used to push the paste in the syringe through the printhead. The extrusion of the paste was conducted at an angle of 90° with a printing platform of 120 × 80 × 50 mm. The nozzle of the printer had a diameter of 0.4 mm.

A model of the single-layer cell was designed in computer-aided design (CAD) software. The model was imported into the Slic3r software as a G-code file. The G-code file contained the information of the print geometry and the other printing parameters such as nozzle diameter, filament diameter, infill of the model, thickness of the printed layer, number of solid/continuous layers, etc. The G-code file was transferred to the printer and executed carefully. The diameter and thickness of the printed cells were set to 17 mm and 2.6 mm, respectively. The printing of a single-layer cell is shown in Figure 2g. After printing, the printed cells were sintered in an oven (shown in Figure 2h) for 2 h at different temperatures ranging from 700 °C to 1000 °C. The heating and cooling rates were set to less than 2 °C per minute. After sintering, the printed cells were placed on a ceramic slab as shown in Figure 2i. Finally, to improve the electrical contact between the single-layer cell and the fuel cell setup, gold paste was applied to both sides of the printed pellets. To set a benchmark for the performance of the printed cells, reference cells were fabricated through a conventional pressing method by pressing the composite powder (Gd_0.2_Ce_0.8_O_2_-Li_2_CO_3_-Na_2_CO_3_-K_2_CO_3_ 60wt%, Li_0.15_Ni_0.45_Zn_0.4_O_2_ 40wt%) with a pressure of 250 MPa in a 13 mm diameter die for 2 min. The gold paste and Ni foam were applied on both sides of the pressed cells to improve their electrical contacts with the measurement setup.

The electrochemical characterizations including the current–voltage (IV) measurements and the electrochemical impedance spectroscopy (EIS) were performed with an electrochemical workstation (Zahner Elektrik IM6, Kronach, Germany). The IV curves were measured with slew rates of 5 to 20 mV per second at 550 °C. The hydrogen gas was supplied to one side of the cell, whereas the air was supplied to the other side of the single-layer cells. The EIS measurements were conducted in an open-circuit condition (OCV) for a frequency range of 100 mHz to 100 kHz using an ac signal amplitude of 20 mV. The Rikagu SmartLab X-ray diffractometer (Tokyo, Japan) was used to characterize the samples, which was equipped with a rotating anode X-ray source (Cu, 9 kW) and a HyPix-3000 detector. The spectra were measured at a scan rate of 6°/min from 2θ = 20° to 2θ = 80°. A high-resolution transmission electron microscope (HR-TEM, Jeol JEM-2800, Akishima, Tokyo, Japan) was used to characterize the particle size distribution and to identify the elements in the composite materials. A field emission scanning electron microscope (FE-SEM, Zeiss Sigma VP, Oberkochen, Germany) was used to observe the morphology of the cells. In addition, another SEM unit (JEOL JSM-7500F, Akishima, Tokyo, Japan), which was coupled with energy dispersive X-ray spectroscopy (EDS), was used to carry out the elemental analysis on the cells. The surface area of the printed and the pressed cells was studied with Brunauer–Emmett–Teller (BET) analysis (Micromeritics TriStar 3020, Norcros, GA, USA). Finally, the short-term stability of the printed cells was tested for 8 h under open-circuit conditions.

## 3. Results and Discussion

### 3.1. XRD and Microscopic Analysis of the Composite Material

**Figure 3:**
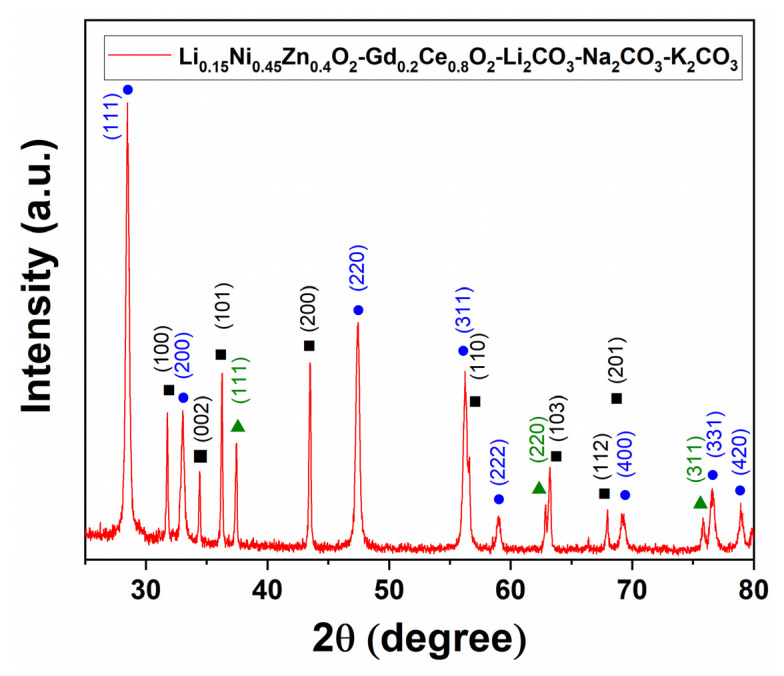
The XRD pattern of the Li_0.15_Ni_0.45_Zn_0.4_O_2_-Gd_0.2_Ce_0.8_O_2_-Li_2_CO_3_-Na_2_CO_3_-K_2_CO_3_ composite material after calcination at 800 °C in air for 3 h. This composite material was used to fabricate all the cells, which were sintered for 2 h at a temperature of 700 °C to 1000 °C. The symbols represent different materials: ● Gd_0.2_Ce_0.8_O_2_, ▲ LiNiO_2_, ■ LiZnO_2_.

The XRD spectra of the composite material (Gd_0.2_Ce_0.8_O_2_-Li_2_CO_3_-Na_2_CO_3_-K_2_CO_3_ 60wt%, Li_0.15_Ni_0.45_Zn_0.4_O_2_ 40wt%) are shown in Figure 3. The peaks that appeared at 2θ = 28.5 (111), 33 (200), 47.4 (220), 56.3 (311), 59 (222), 69.2 (400), 76.6 (331) and 78.9 (420) were identified with the International Center for Diffraction Data (ICDD) database file no. 04-015-2396, which is related to the fluorite lattice structure of the Gd_0.2_Ce_0.8_O_2_. The characteristic peaks at 2θ = 37.4 (111), 62.8 (220) and 75.8 (311) were identified with ICDD file no. 04-006-8078, which is associated with the LiNiO_2_. Similarly, the peaks at 2θ = 31.8 (100), 34.4 (002), 36.3 (101), 43.5 (200), 56.6 (110), 63.2 (103), 67.9 (112) and 69.1 (201) were identified with ICDD file no. 04-013-7260, which is associated with the LiZnO_2_. The identification of these peaks in the spectra confirms the successful synthesis of the composite material for single-layer cell fabrication. The average particle sizes of Gd_0.2_Ce_0.8_O_2_, LiNiO_2_ and LiZnO_2_ were calculated using the Scherrer equation [42] and were found to be 24.8 nm, 53.4 nm and 54.2 nm, respectively.

**Figure 4:**
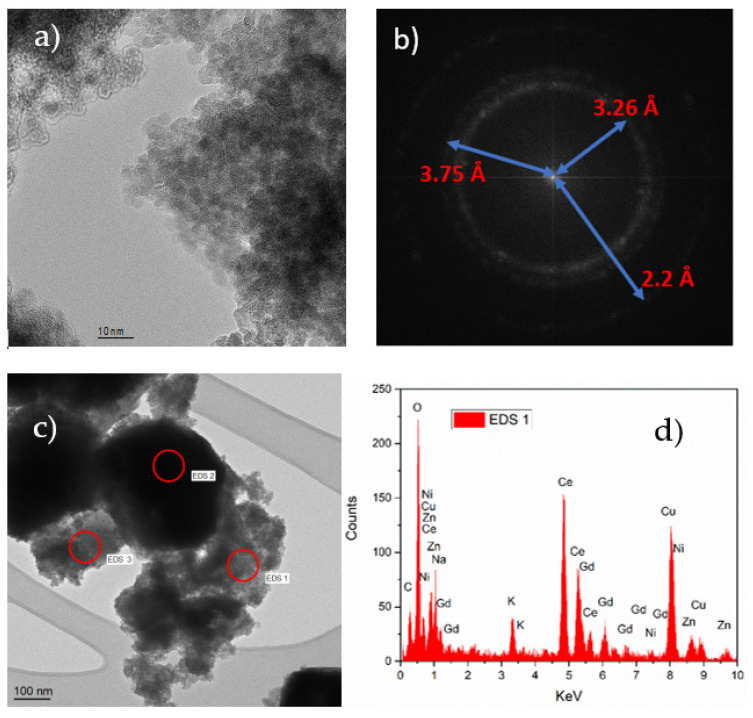
(**a**) High magnification HR-TEM image of the Li_0.15_Ni_0.45_Zn_0.4_O_2_-Gd_0.2_Ce_0.8_O_2_-Li_2_CO_3_-Na_2_CO_3_-K_2_CO_3_ composite material which was in a powder form, (**b**) HR-TEM image after applying FFT filter, (**c**) low-magnification HR-TEM image of the Li_0.15_Ni_0.45_Zn_0.4_O_2_-Gd_0.2_Ce_0.8_O_2_-Li_2_CO_3_-Na_2_CO_3_-K_2_CO_3_ composite powder, (**d**) EDS of the composite powder.

The structures of the composite nanopowder of Li_0.15_Ni_0.45_Zn_0.4_O_2_-Gd_0.2_Ce_0.8_O_2_-Li_2_CO_3_-Na_2_CO_3_-K_2_CO_3_ were analyzed by HR-TEM, as shown in Figure 4. The average size of the particles in the composite material was about 50 nm with a range of 5–90 nm. The atomic lattices of the crystal structures are visible in Figure 4a. In order to find the spacing between the lattice planes, first, a fast Fourier transform (FFT) filter was applied on Figure 4a. Then, masks were applied on the FFT image and, finally, inverse FFT was applied to obtain the spacing between the lattice planes. As illustrated in Figure 4b, different d-spacing values (2.2 Å, 3.26 Å, 3.75 Å) were obtained. It is important to note that the accuracy of the d-spacing values depend on the measurement device calibration. The EDS measurements were conducted at three different spots on the low magnification image shown in Figure 4c. The spectrum of the spot EDS 1 is shown in Figure 4d, which confirms the presence of all the elements in the composite material except Li, i.e., Gd, Ce, O, Na, K, C, Ni and Zn. Due to small size of Li atoms, they cannot be detected in the EDS measurement. The Cu was detected due to the signals from the measurement grid. The EDS spectra collected from the EDS 2 (Figure S1) and EDS 3 (Figure S2) spots are provided in the Supplementary Information.

### 3.2. Electrochemical Characterization

**Figure 5:**
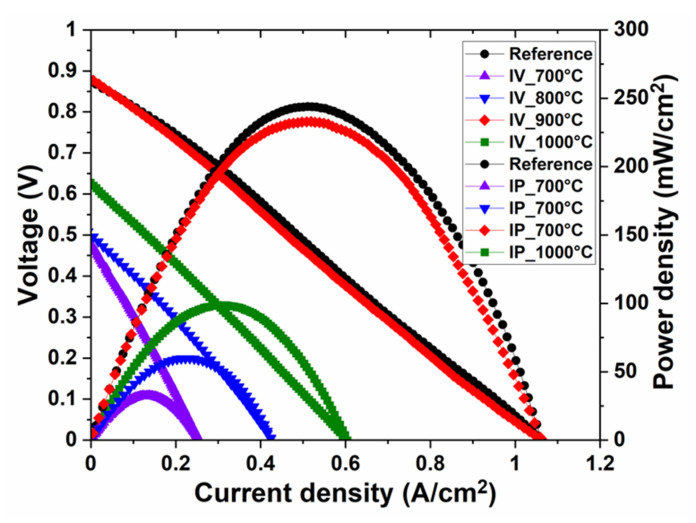
IV and IP curves of the printed single-layer cells sintered at 600 °C, 700 °C, 800 °C, 900 °C and 1000 °C, and the pressed single-layer cell (reference) sintered at 700 °C. All the cells were supplied with H_2_ on one side and air on the other side, with Au and Ni foam serving as the current collector on both sides. These measurements were carried out at 550 °C.

The electrochemical performances of the printed cells and the reference cells were measured at 550 °C while supplying hydrogen gas at one side of the cell and the air at the other side. Figure 5 shows the IV and IP curves of all the printed cells sintered at 700 °C, 800 °C, 900 °C and 1000 °C, and the reference pressed cell sintered at 700 °C. In our previous study, we found that the optimal sintering temperature for pressed single-layer cells is 700 °C [14]; that is the reason, in this study, the pressed cell sintered at 700 °C was chosen as a reference cell. The performance of the printed cells improved gradually with increasing sintering temperature from 700 °C to 900 °C, however, a further increase in the sintering temperature to 1000 °C negatively affected the cell performance. The cells sintered at 700 °C and 800 °C produced only 30 mW/cm^2^ and 60 mW/cm^2^, respectively. The maximum power density of the printed cell sintered at 900 °C dramatically increased to 230 mW/cm^2^, which was quite close to the reference pressed cell. The cell sintered at 1000 °C produced 150 mW/cm^2^. For an optimized performance, a single-layer cell needs to have sufficient density to block the gas crossover. On the other hand, if a single-layer cell is too dense, it would adversely affect the electrode processes, leading to a higher polarization loss in the cell. Another factor which could be detrimental to the cell performance, especially OCV, is electronic leakage through the cell. It is common for single-layer cells to have a lower OCV as compared to conventional three-layer cells, where a distinct electrolyte layer prevents electron leakage. The fabrication method and the thermal treatment given to a single-layer cell could affect both its density and the ability to block an electronic current. Based on the electrochemical results, it is clear that 900 °C is an appropriate sintering temperature for an extrusion-based 3D printed single-layer cell, which provides sufficient density and electronic blocking ability to the cell. A similar electrochemical performance of the pressed single-layer cell was obtained after sintering at 700 °C.

The total cell resistance of the printed cells obtained through the slope of the IV curves decreased with an increase in the sintering temperature as given in Table 1. The printed cell sintered at 900 °C and the reference pressed cell sintered at 700 °C had the same cell resistance of 0.8 Ω·cm^2^. A similar trend was found in the BET surface area of the printed cells, where an increase in the sintering temperature resulted in a reduced BET surface area of the cells. The BET surface areas of the printed cell sintered at 900 °C (0.34 m^2^/g) and the reference cell (0.39 m^2^/g) were quite close to each other. The printed cells sintered at 700 °C and 800 °C had a larger surface area, i.e., 2.93 m^2^/g and 0.73 m^2^/g, respectively, whereas the printed cell sintered at 1000 °C had a smaller surface area (0.18 m^2^/g). It shows that the surface area of a cell significantly affects its electrochemical performance and the optimized performance of the cells requires a certain density of the single layer. It is important to note that the magnitude of the electronic leakage through the single-layer cell could vary depending on the type of electrode material used in it, which could affect its electrochemical performance. Therefore, a systematic study should be performed to monitor the effect of leakage currents through single-layer cells using different electrode materials.

**Figure 6:**
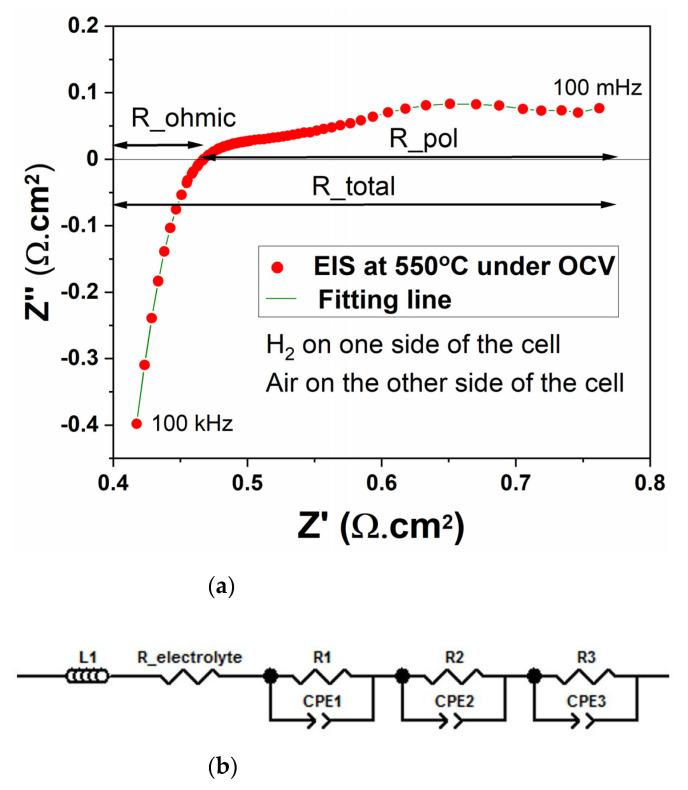
(**a**) The EIS plot of the printed single-layer cell sintered at 900 °C. The measurement was conducted at 550 °C under OCV conditions using a frequency range of 100 mHz to 100 kHz, (**b**) an equivalent circuit model that was used to fit the EIS plot.

The EIS plot of the printed single-layer cell sintered at 900 °C is shown in Figure 6a. An equivalent circuit model shown in Figure 6b was used to fit the EIS data in the Z-view software. The inductance of the measurement setup and the ohmic losses in the cell are represented in the model by L1 and R_ohmic, respectively. The electrode processes in the cell are represented by three pairs of R-CPE, which included kinetics linked to catalytic reactions on both sides of the cell and the mass diffusion. The R1-CPE1 and R2-CPE2 impedances in the cells are caused by charge transfer reactions, whereas the R3-CPE3 impedance is caused by mass transfer processes. The capacitances involved in the oxidation and the reduction charge transfer reactions at the electrodes are given by CPE1 and CPE2, respectively. The other chemical capacitance involved in the gas diffusion process is represented by CPE3. Table 2 gives the values of the resistances obtained by fitting the model parameters.

A large value of L1 (0.59 µH) comes mainly from the long wires used in the measured setup. The value for R_ohmic was obtained through the *x*-axis intercept of the high-frequency data. The printed cell showed a substantial ohmic loss (0.46 Ω·cm^2^), which was caused by ionic transport in the cell as well as the cell’s interface with the experimental equipment. In previous studies [9,21,22], it has been demonstrated that a single-layer cell based on Li_0.15_Ni_0.45_Zn_0.4_O_2_-Gd_0.2_Ce_0.8_O_2_ exhibited both H^+^ and O^2−^ conduction. Furthermore, a systematic study to understand ionic transport in a single-layer cell revealed that H^+^ transport was dominant [9]. The O^2−^ ion travel mainly through oxygen vacancies in the doped ceria. On the other hand, H^+^ ions travel through a hopping mechanism via the protonated oxides in the single layer [9]. The details of the hydrogen oxidation reaction (HOR) and oxygen reduction reaction (ORR) in the Li_0.15_Ni_0.45_Zn_0.4_O_2_-Gd_0.2_Ce_0.8_O_2_-based single-layer cell are given in previous studies [9,21,22]. The values for other resistive elements (R1, R2, R3) in the model were obtained by fitting the three depressed semicircles that appear at the lower frequencies. The polarization loss (0.32 Ω·cm^2^) in the cell was calculated by subtracting the ohmic resistance from the total resistance of the cell. The major proportion of the polarization loss (0.21 Ω.cm) comes from the mass diffusion-related electrode processes in the cell. The total resistance of the cell obtained through EIS was 0.78 Ω·cm^2^, which is very close to the value of the total cell resistance obtained through IV measurements (0.80 Ω·cm^2^), as shown in Table 1. From the EIS data, it is clear that both the ohmic and polarization losses are quite high in the cell. These losses can be reduced by improving the contact between the cell and the measurement setup and adding porous structures on both sides of the cells. Adding pattern on surfaces of the cells directly through 3D printing or with the help of laser scribing could help to reduce the polarization loss in the cell. However, a detailed study is required to find the appropriate settings of the 3D printer and the laser scriber to produce high-performance cells.

### 3.3. Structural and Elemental Analysis of the Printed Cell

**Figure 7:**
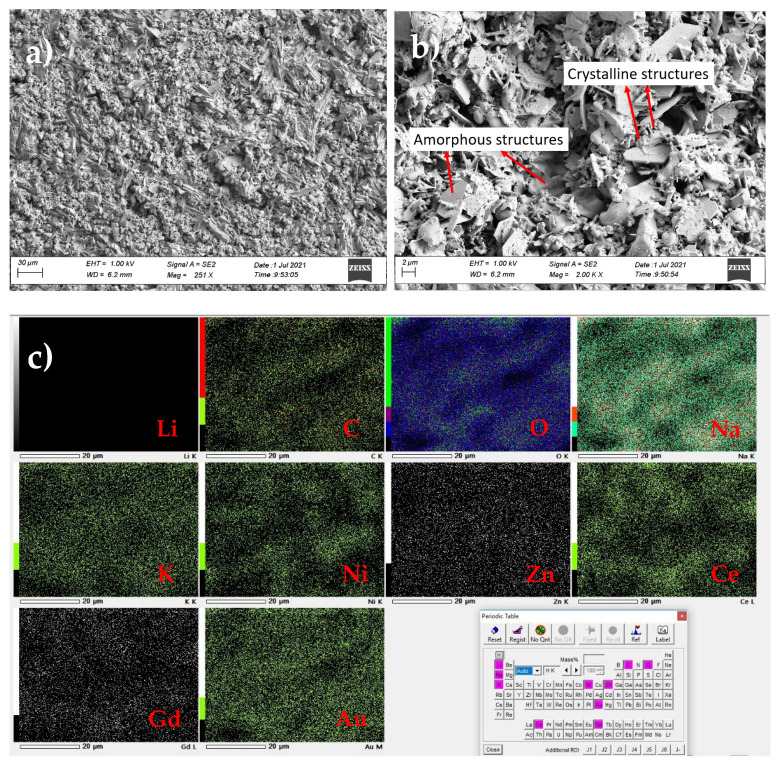
SEM images of the printed single-layer cell sintered at 900 °C: (**a**) at low magnification (251×), (**b**) at high magnification (2000×), (**c**) EDS mapping on the SEM image.

The morphology of the printed cell sintered at 900 °C was investigated through SEM. Figure 7a shows the microstructures in the printed cell at 251X magnification. The surface of the single layer was quite dense and homogenous. Structures of different shapes were observed in the SEM image shown in Figure 7b, which was taken at a higher magnification of 2000×. The grains of varying size (35–220 nm) were observed due to the formation of clusters and milling of the nanopowders in the mixing process during the synthesis of the nanocomposite material. In addition, amorphous structures can be seen in the image due to the presence of alkali-carbonates (Li_2_CO_3_-Na_2_CO_3_-K_2_CO_3_) in the cell. In addition, small needle-like structures were present on the surface of the cell, showing the presence of hydroxides in the cell.

EDS analysis was carried out to identify the elements present in the printed cell sintered at 900 °C. EDS mapping shown in Figure 7c shows the distribution of the elements, including C, O, Na, K, Ni, Zn, Ce, Gd and Au, in the sample. Although Li was present in the cell, it was, however, not detected due to its small size. All the other elements of interest were heterogeneously distributed in the cell. Table 3 shows the elemental concentration on the surface of the cell. Au was present in the sample because Au coating was carried out on the sample before performing the SEM measurements to make the sample sufficiently conductive.

### 3.4. Short-Term Stability Test of the Printed Cell

**Figure 8:**
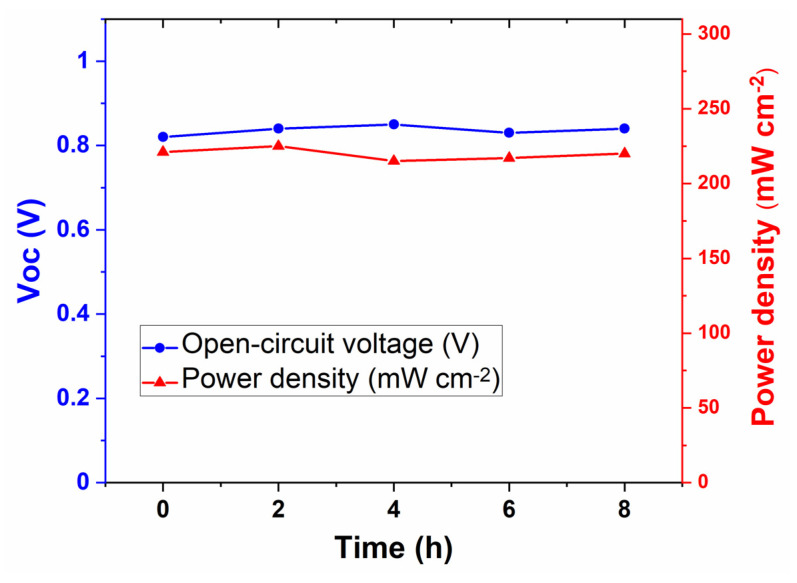
Short-term stability test of the printed single-layer cell sintered at 900 °C under open-circuit conditions. The power density measurements were carried out with an interval of 2 h.

The stability of the printed cell sintered at 900 °C was tested for a short period of time, only 8 h in an open-circuit condition with H_2_ and oxygen as the fuel and oxidant, respectively. IV measurements were carried out in every 2 h to track the cell’s performance deterioration, mainly degradation in the OCV and power density. The short-term stability of the printed cell is shown in Figure 8. The OCV of the cell remained quite steady between 0.82 V and 0.85 V during the test. During the first two hours of operation, the output power density increased from 221 mW/cm^2^ to 225 mW/cm^2^, before stabilizing at 220 mW/cm^2^ after eight hours. It is concluded from this short-term stability test that the composite materials Li_0.15_Ni_0.45_Zn_0.4_O_2_-Gd_0.2_Ce_0.8_O_2_-Li_2_CO_3_-Na_2_CO_3_-K_2_CO_3_ are thermally and chemically compatible and are intrinsically stable materials under cell operating conditions. The long-term stability of the printed cells will be investigated using different fuels, e.g., H_2_, biofuels, etc., in our future work to investigate any possible degradation mechanism in printed fuel cell devices. As far as thermo-mechanical stability is concerned, single-layer printed cells are expected to perform better than conventional three-layer cells which usually suffer from a mismatch in the thermal expansion coefficients (TECs) of the three layers.

## 4. Conclusions

Utilizing the composite Li_0.15_Ni_0.45_Zn_0.4_O_2_-Gd_0.2_Ce_0.8_O_2_-Li_2_CO_3_-Na_2_CO_3_-K_2_CO_3_, it was successfully demonstrated that an extrusion-based 3D printer can be used to fabricate single-layer ceramic fuel cells with the same excellent performance as when using the traditional pressing method. It was observed that the thermal treatment given to cells significantly affected their density, which affected their electrochemical performance. In this systematic study, it was found that the single-layer cells sintered at 900 °C produced the best performance (230 mW/cm^2^), which was quite close to the performance achieved through the conventional pressing method (240 mW/cm^2^). The printed cell’s performance was mainly limited due to high ohmic and polarization losses, which resulted in high total cell resistance. The value of the total cell resistance obtained through IV measurements and the EIS measurement was 0.8 Ω·cm^2^ and 0.78 Ω·cm^2^, respectively. Unlike most of the single-layer ceramic fuel cell literature, in this study, NCAL-coated Ni foams were not applied on the sides of the cell, because this material acts as an electrode in the cell, making it a three-layer cell structure. The disadvantage of the three layers could be reflected in the long-term stability of the cell because of lower thermal stability due to a mismatch in the TECs of the layers. Moreover, extrusion printing is not suitable for depositing thin films (of a few micrometers) in a three-layer cell structure due to the low resolution of extrusion printers. Future research should focus on the long-term durability of printed single-layer cells to better understand their possible degradation mechanisms.

The performance of the 3D printed single-layer ceramic fuel cells could be further improved by introducing pattern structures on both sides of the cells to increase the surface area, which would improve the reaction kinetics as well as the mass transport in the cell. These pattern structures can be printed directly through the 3D printer. However, if the resolution of the 3D printer hinders the printing of small flow channels on the surface of the cell, high-resolution laser scribing could be used as an alternative method to achieve the pattern structures. A hybrid of extrusion-based 3D printing and laser scribing has the potential to produce high-performance single-layer ceramic fuel cells, which should be investigated more in future studies.

## Figures and Tables

**Table 1:** The peak power density and the total cell resistance under fuel cell conditions, and the BET surface area of the printed cells and the reference pressed cell.

Sintering Temperature (°C)	Max. Power Density (mW/cm^2^)	Total Cell Resistance (Ω·cm^2^)	BET Surface Area (m^2^/g)
700	30	2.0	2.93
800	60	1.2	0.73
900	230	0.8	0.34
1000	150	0.7	0.18
Reference	240	0.8	0.39

**Table 2:** Resistance values corresponding to fuel cell processes using the equivalent circuit shown in Figure 6b. The EIS measurements were carried out at 550 °C on a single-layer printed cell sintered at 900 °C with dimensions of 11.5 mm in diameter and a thickness of 1.65 mm.

L1 (µH)	R_ohmic (Ω·cm^2^)	R1 (Ω·cm^2^)	R2 (Ω·cm^2^)	R3 (Ω·cm^2^)	R_pol (Ω·cm^2^)	R_total (Ω·cm^2^)
0.59 ± 0.01	0.46 ± 0.03	0.07 ± 0.02	0.09 ± 0.01	0.21 ± 0.03	0.32 ± 0.04	0.78 ± 0.06

**Table 3:** Elemental concentration of the printed single-layer cell shown in Figure 7. ND represents “no data”.

Element	Li	C	O	Na	K	Ni	Zn	Ce	Gd	Au	Total
Mass (%)	ND	1.58	18.07	2.76	1.63	18.09	6.82	34.57	8.84	7.65	100

## Data Availability

The data presented in this study are available on request from the corresponding author.

## Supplementary Materials

The following are available online at https://www.mdpi.com/article/10.3390/nano11092180/s1, Figure S1: EDS of the composite powder from spot EDS 2 in Figure 4a, Figure S2: EDS of the composite powder from spot EDS 3 in Figure 4a.
