# Supplementary material for: Systematic Analysis on the Effect of Sintering Temperature for Optimized Performance of Li0.15Ni0.45Zn0.4O2-Gd0.2Ce0.8O2-Li2CO3-Na2CO3-K2CO3 Based 3D Printed Single-Layer Ceramic Fuel Cell
Source: Nanomaterials (Basel). 2021 Aug 25;11(9):2180. doi: 10.3390/nano11092180 (PMC8466634; doi:10.3390/nano11092180)
Supplement: Supplementary file 1 [file nanomaterials-11-02180-s001.zip › nanomaterials-1332622-supplementary.pdf]

nanomaterials

MDPI

SUPPLEMENTARY INFORMATION

Systematic analysis on the effect of sintering temperature for optimized performance of $Li_{0.15}Ni_{0.45}Zn_{0.4}O_2$-$Gd_{0.2}Ce_{0.8}O_2$-$Li_2CO_3$-$Na_2CO_3$-$K_2CO_3$ based 3D printed single-layer ceramic fuel cell

Muhammad Imran Asghar [1,2,*], Pyry Mäkinen [1], Sini Virtanen [1], Anna Maitre [1], Maryam Borghei [3] and Peter D. Lund [1]

[1] New Energy Technologies Group, Department of Applied Physics, School of Science, Aalto University, P.O. BOX 15100, FI-00076 Espoo, Finland; imran.asghar@aalto.fi; pyry.makinen@aalto.fi; sini.n.virtanen@aalto.fi; anna.maitre@aalto.fi; peter.lund@aalto.fi

[2] Faculty of Physics and Electronic Science, Hubei University, Wuhan, Hubei, 430062, China; imran.asghar@aalto.fi

[3] Department of Bioproducts and Biosystems, School of Chemical Engineering, Aalto University, FI-00076 Espoo, Finland; maryam.borghei@aalto.fi

* Correspondence: imran.asghar@aalto.fi

Supporting results

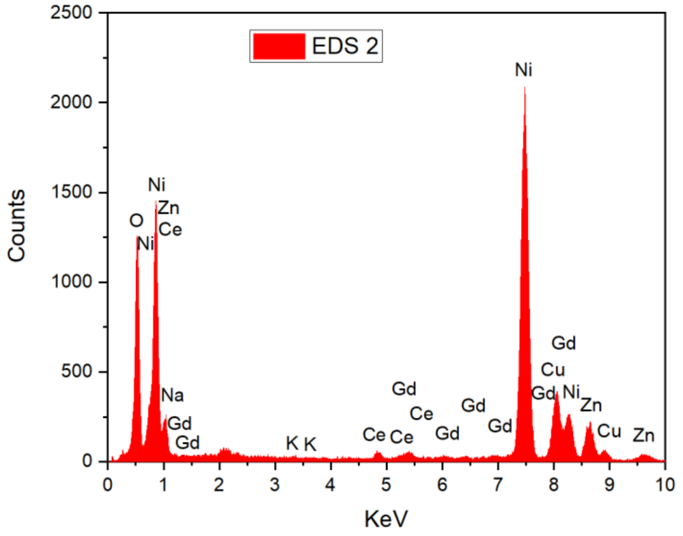

Figure S1. EDS of the composite powder from spot EDS 2 in Figure 4a.

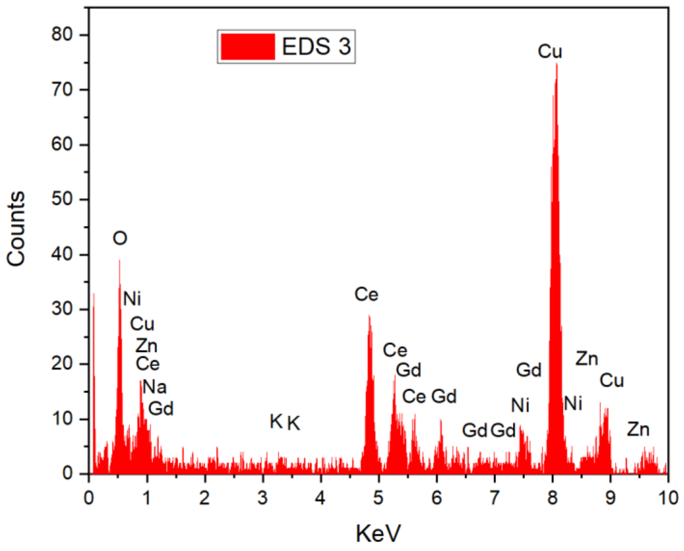

Figure S2. EDS of the composite powder from spot EDS 3 in Figure 4a.
